# Preparation of the 1-Methylimidazole Borane/Tetrazole System for Hypergolic Fuels

**DOI:** 10.3390/molecules27144466

**Published:** 2022-07-13

**Authors:** Xue Li, Jun Wu, Fan Fang, Hongping Li, Lei Wang, Hui Wan, Guofeng Guan

**Affiliations:** 1 State Key Laboratory of Materials-Oriented Chemical Engineering, Jiangsu National Synergetic Innovation Center for Advanced Materials, College of Chemical Engineering, Nanjing Tech University, Nanjing 210009, China; lx158618@163.com (X.L.); wujun20180329@163.com (J.W.); wanhui@njtech.edu.cn (H.W.); 2 Centre Hydrogenergy, College of Material Science & Technology, Nanjing University of Aeronaut & Astronaut, Nanjing 210016, China; fangfan1990@nuaa.edu.cn; 3 Institute for Energy Research of Jiangsu University, Jiangsu University, Zhenjiang 212013, China; hongpingli@ujs.edu.cn

**Keywords:** borane, azole, hypergolic fuel, acid–base neutralization

## Abstract

Based on the acid–base neutralization, the (1-methylimidazolium)(tetrazol-1-yl)borane was successfully synthesized by taking advantage of the acidity of the tetrazole and the basicity of the 1-methylimidazole borane complex. Through HRMS, NMR, and FT−IR, the structure of synthetic compounds was characterized in detail. Concerning about the (1-methylimidazolium)(tetrazol-1-yl)borane, it had an ignition−delay time of about 25 ms and a density specific impulse over 351 s·g/cm^3^, making it a suitable candidate for green hypergolic fuels. Moreover, it also demonstrated that introducing tetrazole into the borane could be an appropriate strategy to adjust the performance of the energy of those borane compounds.

## 1. Introduction

Hydrazine and its derivatives are often used as parts of bipropellants in rockets, resulting from the low cost and simple synthetic routes. However, with the large-scale utilization of hydrazine and its derivatives, pollutions to environment and carcinogenicity to humans also spring up. Therefore, special care must be taken in handling such materials, which makes green hypergolic propellants draw significant attention [1].

**Figure 1:**
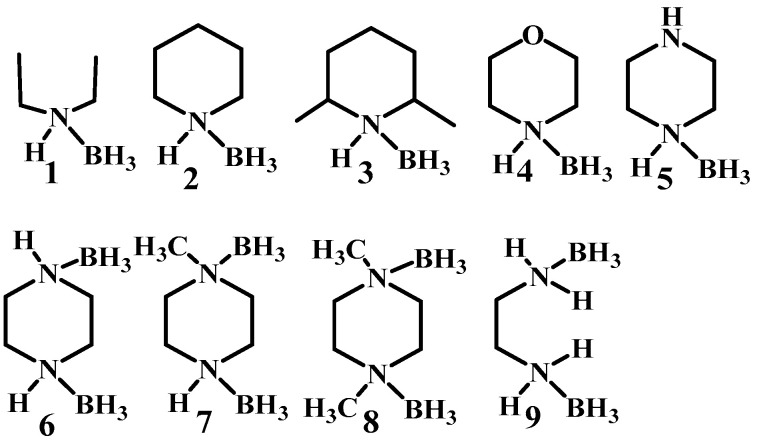
The structure of amine-boranes and diamine-bisboranes [13].

Boron has been involved in the hypergolic bipropellant for its high calorific value and strong reducibility for improving the performance of the ignition delay times and specific impulse [2,3,4]. In order to take advantage of the unique properties of boron, a series of compounds containing B–H bonds are taken into consideration utilizing electrophilic B–H substitution by [5,6,7,8]. As the simplest B–H containing compound, the borane has attracted much interest. In most cases, the borane applied in the hypergolic bipropellants, such as decaborane and diborane exhibit high toxicity, making it a new challenge concerning the utilization of the B–H bonds. In fact, the borane can not only exist as the caged borane but can also be present in the form of complexes, such as THF·BH_3_ and NH_3_·BH_3_. Thus, heterocycles combing with boron can be synthesized by utilizing the empty orbital of boron and the lone pair of electrons of heteroatoms in heterocyclic compounds [9,10,11] for potential fuels applied in bipropellants. Plenty of borane complexes have been successively developed based on this strategy. Baier et al. tested the ignition-delay (ID) times of the ammonia borane (AB) with 100% HNO_3_ under different conditions. All of them had an ID time around 2–10 ms, which could compete with that of the unbalanced dimethylhydrazine (UDMH, 4.8 ms) [12]. By replacing the ammonia with saturated heterocyclic compounds, the same groups developed new types of ammonia borane, as shown in Figure 1. Most synthesized AB still had an ID time below 10 ms, proving that ammonia borane may be a good alternative for hydrazine and its derivatives [13].

**Figure 2:**
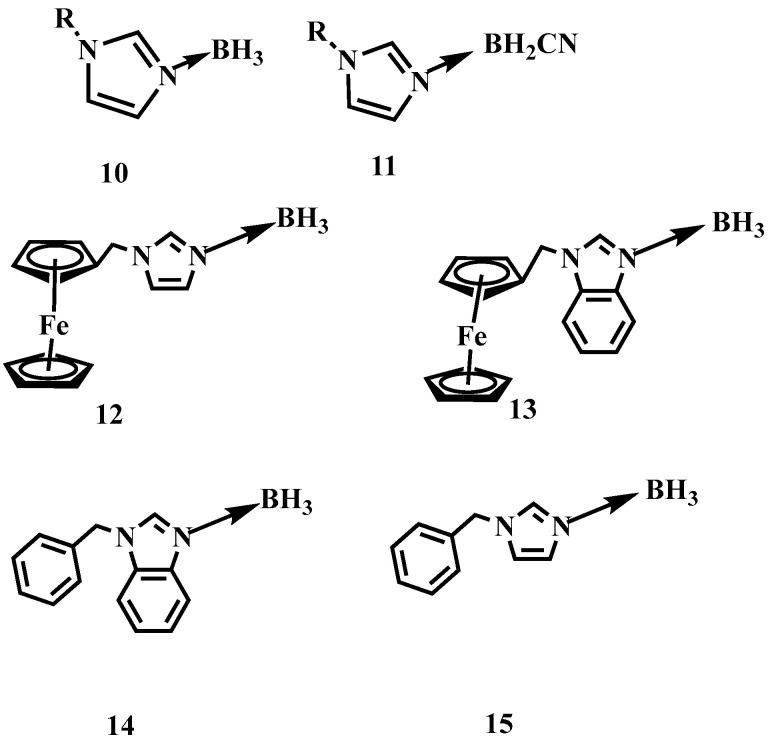
The structure of *N*-alkyl imidazole borane, *N*-alkyl imidazole cyanoborane, *N*-(arylmethylene)-benzimidazole borane and *N*-(arylmethylene)-imidazole borane [14,15,16].

To improve the enthalpy of the formation (∆H_f_) and combustion (∆H_c_) of such AB based materials, a series of *N*-alkyl imidazole borane or cyanoborane complexes have been prepared by Zhang and Chen et al. [14,15]. By substituting ammonia with *N*-alkyl imidazole, the *N*-alkyl imidazole borane or cyanoborane complex obtained values of ∆H_f_ higher than −0.51 or 0.22 kJ/g, which is far higher than that of the AB (−2.17 kJ/g). Yuan et al. further employed the *N*-(arylmethylene)-benzimidazole and *N*-(arylmethylene)-imidazole as the electron donor to coordinate with BH_3_, as shown in Figure 2 [16]. Although the synthesized substance is solid at room temperature, they still have good performance of ignition delay times. Some of them were only 5 ms.

**Figure 3:**
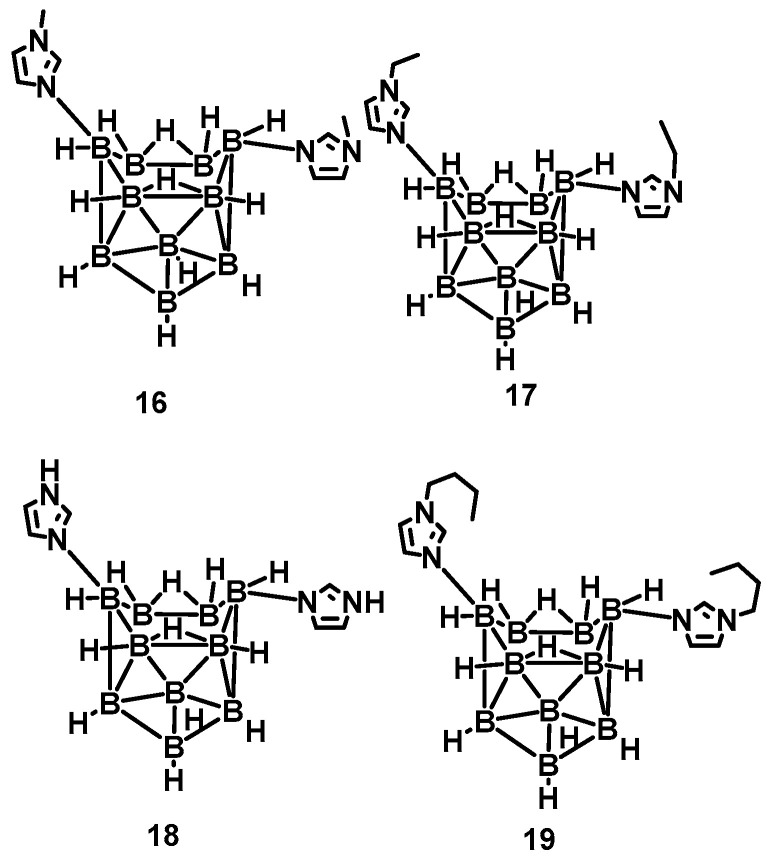
The structure of *N*-alkyl imidazole-B_10_H_12_ and imidazole-B_10_H_12_ complexes [20].

Nevertheless, the above synthetic strategies are mainly based on the substitution of ammonia, thereby ignoring the reactivity of BH_3_ in the borane [17,18,19]. Generally speaking, the BH_3_ group is often used as an active site to participate in organic reactions. Rogers et al. utilized the basicity of imidazole, *N*-alkyl imidazole, and the acidity of the B-6 and B-9 in the n-B_10_H_14_ [20]. Imidazole-substituted nido-decaboranes were also prepared, as shown in Figure 3. The remarkable hypergolicity caused these compounds to have an ID time of only 2 ms, even blended with MeOH, EtOH, and THF. All the mixtures held an ID time below 50 ms, which met the requirements for application. Besides, the same team proposed a cocrystal-based strategy by utilizing the imidazole-substituted nido-decaborane as the hypergolic trigger, co-crystallized with the nitrobenzene to promote the performance of the ∆H_c_, enhancing enthalpy of combustion from −11,815 to −14,993 kJ/mol [21]. This means introducing the azole in the borane could make the synthesized substance own a considerable improvement in the performance of the energy.

**Figure 4:**
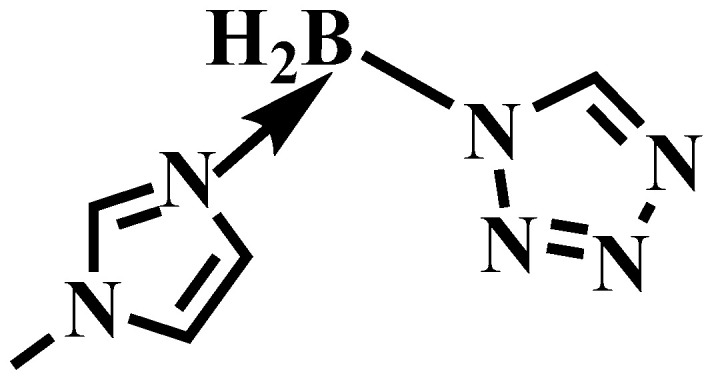
The structure of (1-methylimidazolium)(tetrazol-1-yl)borane.

Herein, for the sake of improving the ∆H_f_ and ∆H_c_ of the 1-methylimidazole borane complex, taking the acidity of the tetrazole and the basicity of the 1-methylimidazole borane complex into an advantage, the (1-methylimidazolium)(tetrazol-1-yl)borane was successfully synthesized by acid–base neutralization, as shown in Figure 4. The mixtures were also prepared for real-life applications by blending 1-methylimidazole borane complex with different mass fractions of tetrazole. Through HRMS, NMR, and FT-IR, the structure of the synthetic compound was fully characterized. DSC and TGA were conducted to test the physicochemical properties of the compounds and the mixtures. ID test was implemented to find the differences in ignition performance. ∆H_f_, ∆H_c_, vacuum specific impulse (I_vac_), and density specific impulse (ρI_sp_) were all calculated to verify the influence of the addition of the tetrazole in the performance of energy by Gaussian and NASA-CEA programs. Density functional theory was adopted to investigate the ID times, reaction products, and thermodynamic properties.

## 2. Results and Discussion

### 2.1. NMR, FT-IR, EA

**Figure 5:**
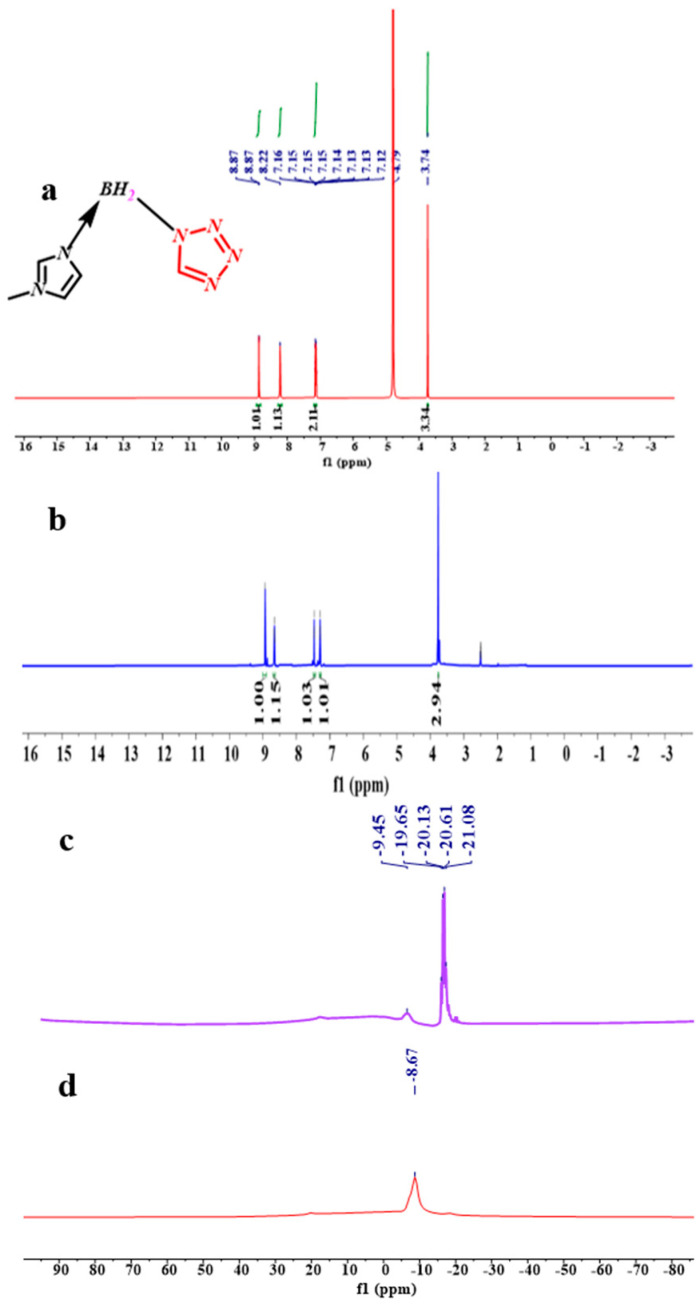
^1^ H NMR spectrum of the (1-methylimidazolium)(tetrazol-1-yl)borane (D_2_O) (**a**); ^1^H NMR spectrum of the (1-methylimidazolium)(tetrazol-1-yl)borane (DMSO-d_6_) (**b**); ^11^B NMR spectrum of 10 wt% tetrazole into 1-methylimidazole borane complex (**c**); ^11^B NMR spectrum of equimolar 1-methylimidazole borane complex and tetrazole (**d**).

**Figure 6:**
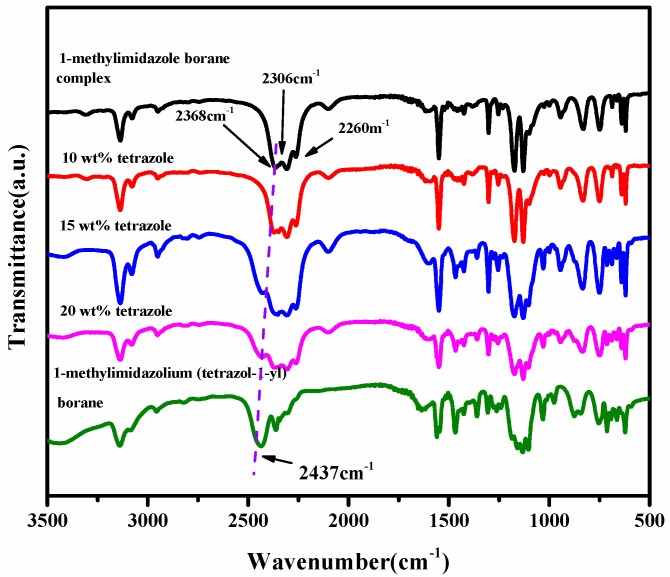
FT-IR of tetrazole with different mass ratios in 1-methylimidazole borane complex and (1-methylimidazolium)(tetrazol-1-yl) borane.

From the spectra of the ^1^H NMR of the (1-methylimidazolium)(tetrazol-1-yl) borane, as shown in Figure 5, the signal of the carbon and hydrogen atoms in the tetrazole can be found, which proves the existence of the structure of the tetrazole in the (1-methylimidazolium)(tetrazol-1-yl) borane when comparing the spectra of the ^1^H NMR and ^13^ CNMR about the 1-methylimidazole and final product, as shown in Figures S1–S3. In order to verify the composition of the (1-methylimidazolium)(tetrazol-1-yl)borane, elemental analysis was also carried out. It seems the result of C/N was nearly same as that based on the calculation. Thanks to the chemical environment of boron in the (1-methylimidazolium)(tetrazol-1-yl) borane being different from that of the 1-methylimidazole borane complex, the ^11^B NMR was chosen to operate. As shown in Figure 5, it can be found that when the tetrazole and 1-methylimidazole borane complex were in equal molar ratio, in the ^11^B NMR spectrum, only a single signal is found at −8.67 ppm, which shall be ascribed to the structure of the BH_2_. On the contrary, when the addition of tetrazole into the 1-methylimidazole borane complex was 10 wt%, the prominent peaks in the ^11^B NMR spectrum located around −20 ppm, which is attributed to the BH_3_ in the 1-methylimidazole borane complex. Then, to further demonstrate the existence of the B–H in the final structure, the FT-IR was also conducted. As shown in Figure 6, with additions of tetrazole, a peak near 2430 cm^−1^ in the IR spectra gradually emerges, which shall be assigned to the structure of the BH_2_. Therefore, when the 1-methylimidazole borane complex reacts with the tetrazole with equimolar, the main area in the IR spectra located at the 2300–2500 cm^−1^, only has a peak around 2435 cm^−1^, which further confirms the existence of the BH_2_ rather than BH_3_ in the product.

### 2.2. Density and Viscosity

**Figure 7:**
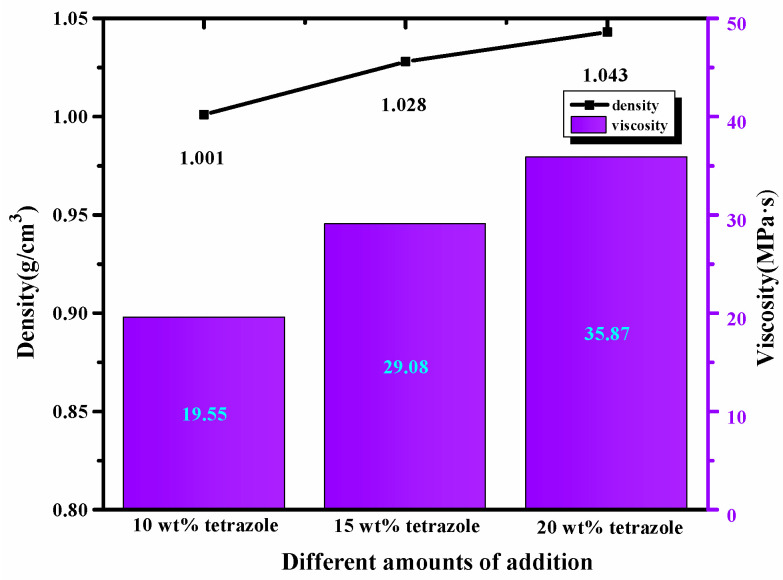
Density and viscosity of the different mass ratios additions in the 1-methylimidazole borane complex.

High density was preferred in choosing the fuels, considering the density’s importance in the fuel. Compared with the 1-methylimidazole borane complex, (1-methylimidazolium)(tetrazol-1-yl)borane density was 1 g/cm^3^ higher than 0.90 g/cm^3^ of the mentioned borane complex, which was further beneficial for improving the performance of the density specific impulse. In addition, for the mixtures, with the addition of the tetrazole, all of them also obtained a higher density than the initial 1-methylimidazole borane complex. Meanwhile, the value of the density was also consistent with the addition of azole. Furthermore, with the amount of tetrazole increases, the viscosity of the mixtures also showed the same trend as the density. Meanwhile, as shown in Figure 7, although the addition of the tetrazole increased the viscosity of the mixtures to some degree, the final viscosity value was still in an appropriate range for the application.

### 2.3. Thermal Properties

**Figure 8:**
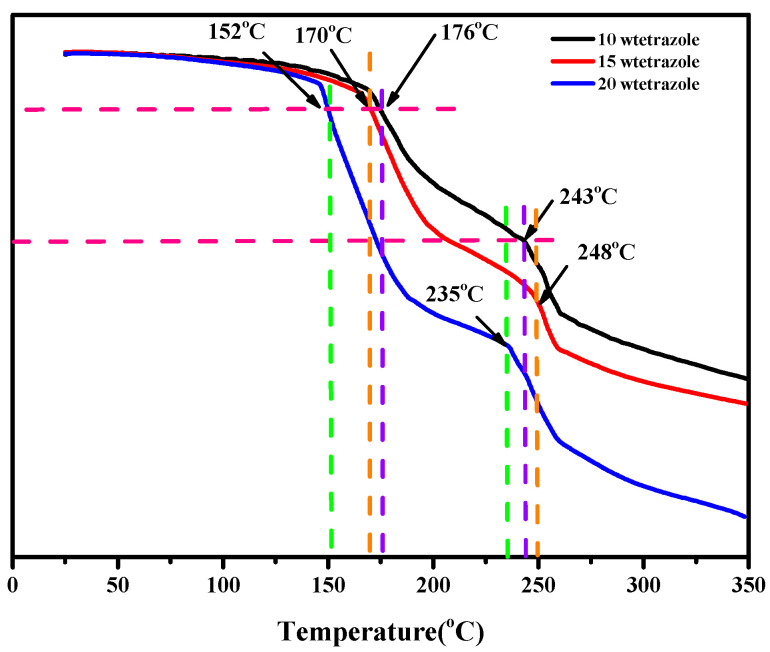
TGA of the different mass ratios additions of tetrazole in the 1-methylimidazole borane complex.

The thermal properties of the compounds and the mixtures were conducted through the TGA and DSC. Comparing the results of DSC and TGA of the (1-methylimidazolium)(tetrazol-1-yl)borane, as shown in Figures S4 and S5. We found the mentioned product had a thermal decomposition temperature (T_d_) up to 190 °C, which may result from the strong boron-nitrogen bond in the (1-methylimidazolium)(tetrazol-1-yl)borane, according to the analysis of interaction region indicator (IRI). Then, with regard to the mixtures, all of them had a Td over 150 °C, as shown in Figure 8, which was higher than that of the UDMH.

### 2.4. Heat of Formation and Combustion

**Figure 9:**
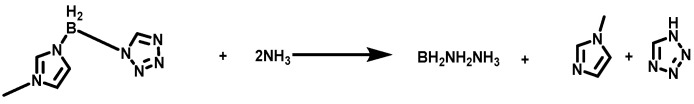
Isodesmic reaction of the (1-methylimidazolium)(tetrazol-1-yl)borane.

**Figure 10:**
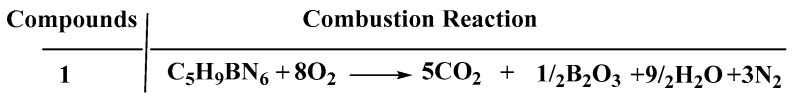
Enthalpy of combustion reaction of the (1-methylimidazolium)(tetrazol-1-yl)borane.

Concerning calculation of the heat of the combustion (∆H_c_) and formation (∆H_f_), the enthalpy of atomization based on the G2 methods and the isodesmic reaction were adopted to investigate that in detail, which were shown in the Supporting information in Tables S1 and S2 [22]. As shown in Figure 9 and Figure 10, the combustion reaction was taken into consideration to evaluate the ∆H_c_ accurately. Compared with the 1-methylimidazole borane complex, (1-methylimidazolium)(tetrazol-1-yl)borane obtained a higher value of ∆H_f_ (214 kJ/mol), far higher than that of 1-methylimidazole borane complex which was 15.39 kJ/mol. Moreover, a higher value of ∆H_f_ was also beneficial for the specific impulse of the propellants and more appreciated for the usage.

### 2.5. Specific Impulse and Density Specific Impulse

**Figure 11:**
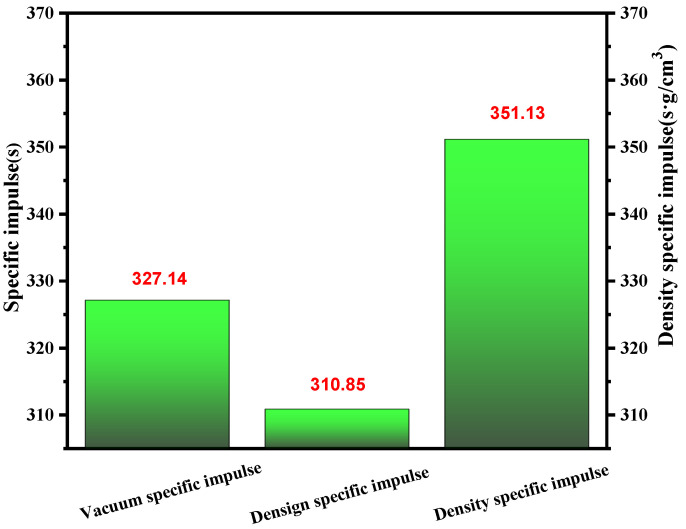
Vacuum density specific impulse, design specific impulse and vacuum specific impulse of (1-methylimidazolium)(tetrazol-1-yl)borane.

When considering the chosen propellants, specific impulse (Isp) was often an essential factor. In most cases, the specific impulse of the propellants should be comparable to that of unsymmetrical dimethyl hydrazine (UDMH). As shown in Figure 11, for the (1-methylimidazolium)(tetrazol-1-yl)borane, it had a value over 320 for vacuum density specific impulse. Then, considering the density, it obtained a density-specific impulse far higher than UDMH, which was limited by its low density. So, the synthesized (1-methylimidazolium)(tetrazol-1-yl)borane met the demands of the user based on the specific impulse to some degree.

### 2.6. ID Times

**Figure 12:**
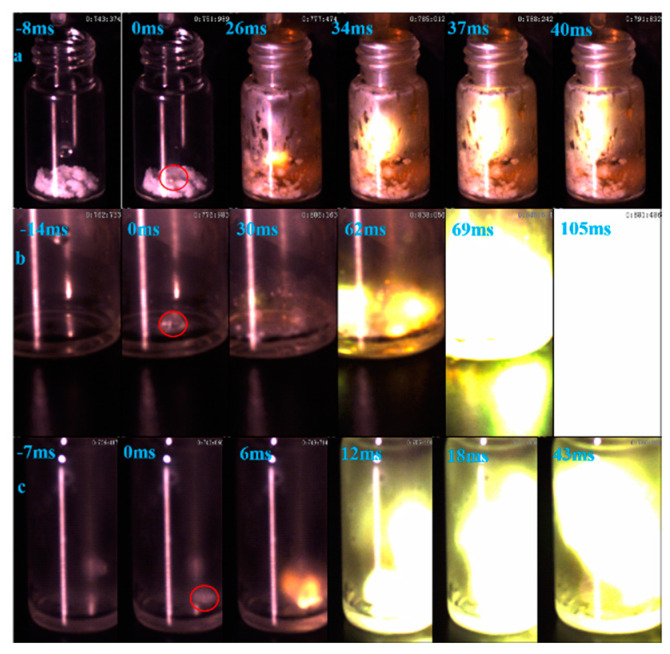
ID of (1-methylimidazolium)(tetrazol-1-yl)borane (**a**); 1-methylimidazole borane (**b**); 20 wt% tetrazole in 1-methylimidazole borane complex (**c**).

**Figure 13:**
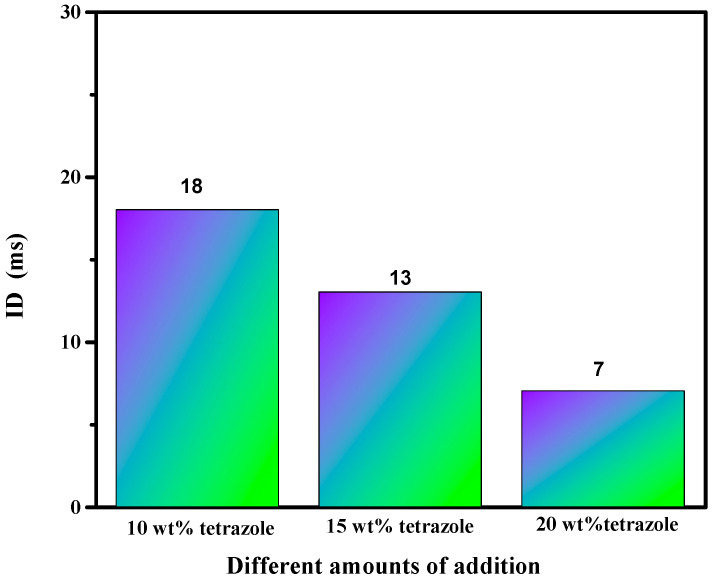
ID time of mixtures.

Ignition-delay time was carried on the high-speed camera at about 1000 fps with 96% HNO_3_. For the (1-methylimidazolium)(tetrazol-1-yl)borane, as shown in Figure 12, the (1-methylimidazolium)(tetrazol-1-yl)borane even had a shorter ID time than the 1-methyl imidazole borane complex. Meanwhile, for the mixtures, with the increase in the addition, the blending based on tetrazole obtained an improvement of the ID times, which may be ascribed to the more containing of the (1-methylimidazolium)(tetrazol-1-yl)borane in the mixtures. Overall, as shown in Figure 13, all the mixtures held ID times less than 50 ms, which nearly met the needs of the application [23].

### 2.7. DFT Calculations

All the calculations were carried out on the Gaussian 09 with Multiwfn 3.8. B3lyp/6-311++G**//M062x/def2tzvp with empirical dispersion, which was chosen to exhibit the interaction region indicator (IRI). B3lyp/6-311++g** is a robust functional basis set widely used in different situations. Especially for organic compounds, the b3lyp/6-311++G** may also be good enough to describe weak interactions when the empirical dispersion (GD3) was applied, as developed by Grimme [24,25]. The results of geometries optimization should be the true minima, and there was no imaginary frequency in the frequency analysis results. Concerning about the average local ion energy (ALIE) and electrostatic potential (ESP) mapping. VMD 1.9.3 was also utilized as the visualization software to display the final results [26,27,28,29,30].

**Figure 14:**
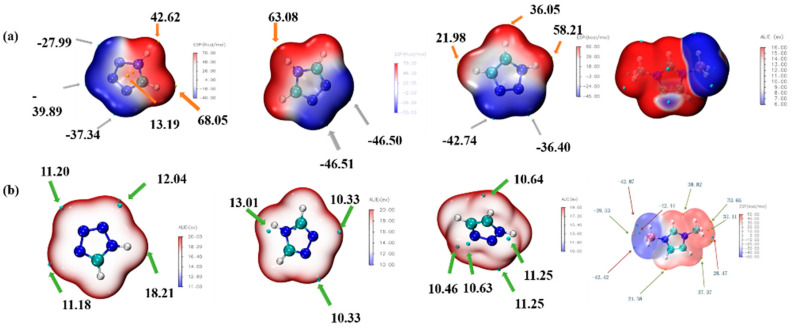
Electrostatic potential mapping (ESP) of 1,2,3,4-tetrazole, 1,2,4-triazole; 1,2,3-triazole and 1-methylimidazole borane complex (**a**); Average local ionization energy mapping (ALIE) of 1,2,3,4-tetrazole, 1,2,4-triazole, 1,2,3-triazole and 1-methylimidazole borane complex (**b**).

**Figure 15:**
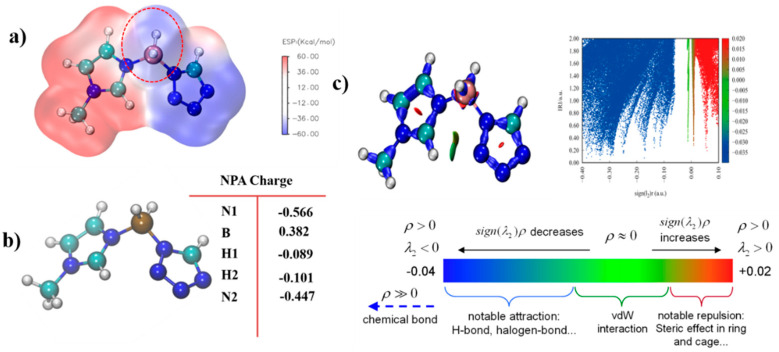
Electrostatic potential mapping of (1-methylimidazolium)(tetrazol-1-yl) borane (**a**); Natural population analysis charges (NPA charge) of the (1-methylimidazolium)(tetrazol-1-yl)borane (**b**); Interaction region indicator (IRI) analysis of the (1-methylimidazolium)(tetrazol-1-yl) borane (**c**).

ALIE is an excellent method to discuss the possible area which would react with the electricity-rich group. In these reactions mentioned above, the BH_3_ group was the most electricity-rich group, which could react with positively charged regions, such as the N–H in the triazole or the tetrazole, which was quantified as displayed by the maximum points of the ESP mapping [31,32,33,34]. As shown in Figure 14, the area surrounding N–H in the tetrazole obtained the maximum value of the ESP mapping, which might be the most possible region among these azoles to react with the BH_3_ group in the 1-methylimidazole borane complex. At the same time, our attempt to obtain (1-methylimidazolium)(triazol-1-yl)borane failed when the triazoles were chosen for their weak electrophilicity. Furthermore, the IRI was also conducted to display the intramolecular forces of matter, which was exhibited in Figure 15, it had a steric hindrance owing to the azole ring. Then, a significant force between the two boron–hydrogen bridged molecular fragments was also included. Owing to the results of the IRI, the (1-methylimidazolium)(tetrazol-1-yl)borane showed spikes (sign(I2)ρ < −0.04) in the scatter plot, which meant a chemical bond rather than weak interaction between the B−N in the (1-methylimidazolium)(triazol-1-yl)borane, which further demonstrated the strong thermal stability by the differential scanning calorimetry (DSC). Moreover, as shown in Figure 15, the negatively charged regions in the ESP mapping of the (1-methylimidazolium)(tetrazol-1-yl)borane should be responsible for the reducibility of this matter. It was also confirmed by the natural population analysis charges (NPA) that all hydrogen atoms in the B–H bonds had negative charges, which meant that the (1-methylimidazolium)(tetrazol-1-yl)borane could react with the 96% HNO_3_, accompaned with a visibly hypergolic phenomenon.

## 3. Experiment Section

### 3.1. Materials and Methods

All the raw materials were purchased from commercial sources (Aladdin, Shanghai, China), and FT-IR was conducted on the WQF-510A, while NMR was operated on the BRUKER ADVANCE. DSC and TG were tested at 5 °C/min on TGA5500 and TAQ2000 NETZSCH, where the sample mass was about 5 mg for DSC and TG. The alumina crucible with argon at 20 mL/min was used to obtain the final results.

### 3.2. Synthesis of (1-Methylimidazolium)(tetrazol-1-yl)borane

First, the 1-methylimidazole borane complex (10 mmol) was dissolved in the toluene (20 mL). Then, tetrazole (10 mmol) was slowly added into the round-bottom flask. The temperature was maintained at 110 °C for 8 h. After that, the solution was divided into two layers, with the temperature cooling down to room temperature. The toluene was removed carefully, while the remaining solvent in the lower layers could be further purified through the rotatory evaporator under reduced pressure. Then, by washing the lower layer with ethyl acetate (50 mL) three times, a white solid product could be obtained, with a yield of 77%, as shown in Figure S6. ^1^H NMR (400 MHz, Deuterium Oxide) δ 3.74 (s, 3H), 7.14 (dt, 2H), 8.22 (s, 1H), 8.87 (s, 1H). ^1^H NMR (400 MHz, DMSO-d_6_) δ 3.77 (s, 3H), 7.29 (t, 1H), 7.47 (t, 1H), 8.66 (d, 1H), 8.93 (s, 1H). ^11^B NMR (193 MHz, DMSO-d_6_) δ −9.84. ^13^C NMR (151 MHz, Deuterium Oxide) δ 34.52, 122.81, 125.06, 138.43, 147.77. HRMS-APCl for C_5_H_10_BN_6_^+^: Calculated 166.10545, Found 166.10545 (Figure S7). Elemental analysis: Calculated C 36.62, H 5.53, N 51.25; Found C 36.51, H 5.57, N 51.10.

### 3.3. Synthesis of Mixtures Based on the Tetrazole and 1-Methylimidazole Borane Complex

#### 3.3.1. 20 wt% Tetrazole

First, the 1-methylimidazole borane complex (0.80 g) was dissolved in the toluene (5 mL). Then, tetrazole (0.20 g) was slowly added to the round-bottom flask. The temperature was maintained at 110 °C for 8 h. After that, the solution was divided into two layers, with the temperature dropping slowly to room temperature. The upper layers should be removed carefully. The remaining solvent in the product could be further purified through the rotatory evaporator under reduced pressure to obtain the aimed viscous, transparent liquids.

#### 3.3.2. 15 wt% Tetrazole

First, the 1-methylimidazole borane complex (0.85 g) was dissolved in the toluene (5 mL). Then, tetrazole (0.15 g) was slowly added into the round-bottom flask. The rest of the process was as mentioned above.

#### 3.3.3. 10 wt% Tetrazole

First, the 1-methylimidazole borane complex (0.90 g) was dissolved in the toluene (5 mL). Then, tetrazole (0.10 g) was slowly added into the round-bottom flask. The rest of the process was as mentioned above.

## 4. Conclusions

In conclusion, (1-methylimidazolium)(tetrazol-1-yl)borane was successfully synthesized through acid–base neutralization. For further practical application, the mixtures by blending 1-methylimidazole borane complex with different mass fractions of tetrazole were also prepared. Among the synthesized compounds, the ΔH_f_ and I_vac_ of (1-methylimidazolium)(tetrazol-1-yl)borane was about 214 kJ/mol and 327.14, far higher than that of 1-methylimidazole borane complex, which was 15.39 kJ/mol and 303.9. In addition, the ESP mapping and NPA charges proved that the negative B–H bonds in the (1-methylimidazolium)(tetrazol-1-yl)borane should be responsible for the hypergolicity. Meanwhile, introducing tetrazole into the borane was proved as an appropriate strategy to adjust the performance of the energy of such borane compounds. Concerning the mixtures of the 1-methylimidazole borane complex and tetrazole, the results of the ID times and density also demonstrated the role that tetrazole played in promoting hypergolicity and performance in terms of energy.

## Data Availability

The data presented in this study are available in Supplementary Materials.

## Supplementary Materials

The following supporting information can be downloaded at https://www.mdpi.com/article/10.3390/molecules27144466/s1, Figure S1: ^13^C NMR of the (1-methylimidazolium)(tetrazol-1-yl)borane; Figure S2: The ^1^H NMR spectrum of the 1-methylimidazole borane (a) and (1-methylimidazolium)(tetrazol-1-yl)borane (b); Figure S3: The ^13^C NMR spectrum of the (1-methylimidazolium)(tetrazol-1-yl)borane (a) and 1-methylimidazole borane (b); Figure S4: DSC of (1-methylimidazolium)(tetrazol-1-yl)borane, Figure S5: TGA of the (1-methylimidazolium)(tetrazol-1-yl)borane; Figure S6: The state of the 1-methylimidazole borane (a), (1-methylimidazolium)(tetrazol-1-yl)borane (b), 1-methylimidazole borane/tetrazole mixtures (10 wt%, 15 wt%, 20 wt%) (c); Figure S7: HRMS-APCl spectra of (1-methylimidazolium)(tetrazol-1-yl)borane; Table S1: Calculated data of gas-phase formation enthalpy based on isodesmic reaction; Table S2: Gas-phase formation enthalpy data used in the isodesmic reaction.

## Sample Availability

Samples of the compounds are available from the authors.
