# Supplementary material for: Preparation of the 1-Methylimidazole Borane/Tetrazole System for Hypergolic Fuels
Source: Molecules. 2022 Jul 13;27(14):4466. doi: 10.3390/molecules27144466 (PMC9323667; doi:10.3390/molecules27144466)
Supplement: Supplementary file 1 [file molecules-27-04466-s001.zip › molecules-1786181-supplementary.pdf]

molecules

MDPI

Supporting Information

Preparation of the 1-Methylimidazole Borane/Tetrazole System for Hypergolic Fuels

Xue Li[1], Jun Wu[1], Fan Fang[2], Hongping Li[3], Lei Wang[1,*], Hui Wan[1], Guofeng Guan[1,*]

1. State Key Laboratory of Materials-Oriented Chemical Engineering, College of Chemical Engineering, Jiangsu National Synergetic Innovation Center for Advanced Materials, Nanjing Tech University, Nanjing 210009, P. R. China.
2. Centre for Hydrogenergy, College of Materials Science and Technology, Nanjing University Aeronautics,Nanjing,210016, China.
3. Institute for Energy Research of Jiangsu University, Jiangsu University, Jiangsu 212013, China

***Corresponding authors:**

Lei Wang, Tel.: +86-25-83587198, E-mail: wanglei@njtech.edu.cn

Guofeng Guan, Tel.: +86-25-83587198, E-mail: guangf@njtech.edu.cn

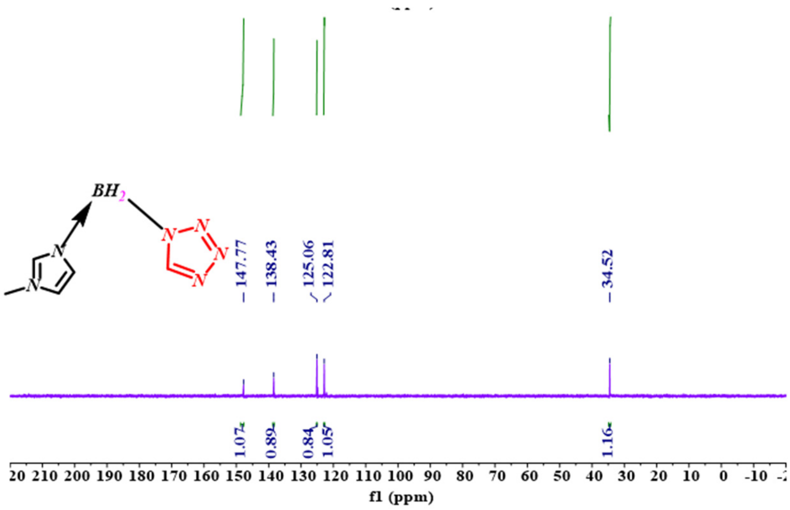

Figure S1. ^{13}C NMR of the (1-methylimidazolium)(tetrazol-1-yl)borane.

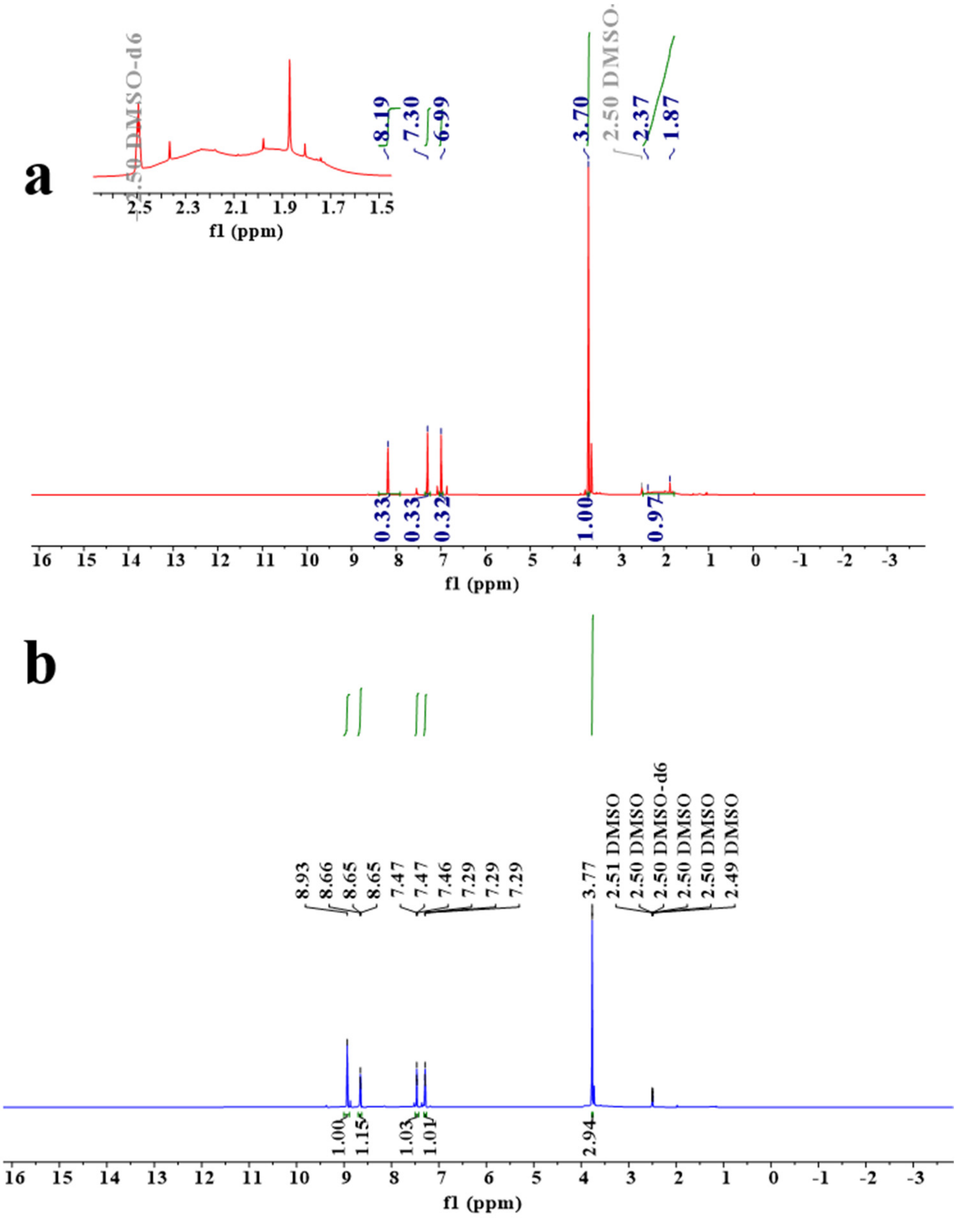

Figure S2. The ^{1}H NMR spectrum of the 1-methylimidazole borane (a) and (1-methylimidazolium)(tetrazol-1-yl)borane (b).

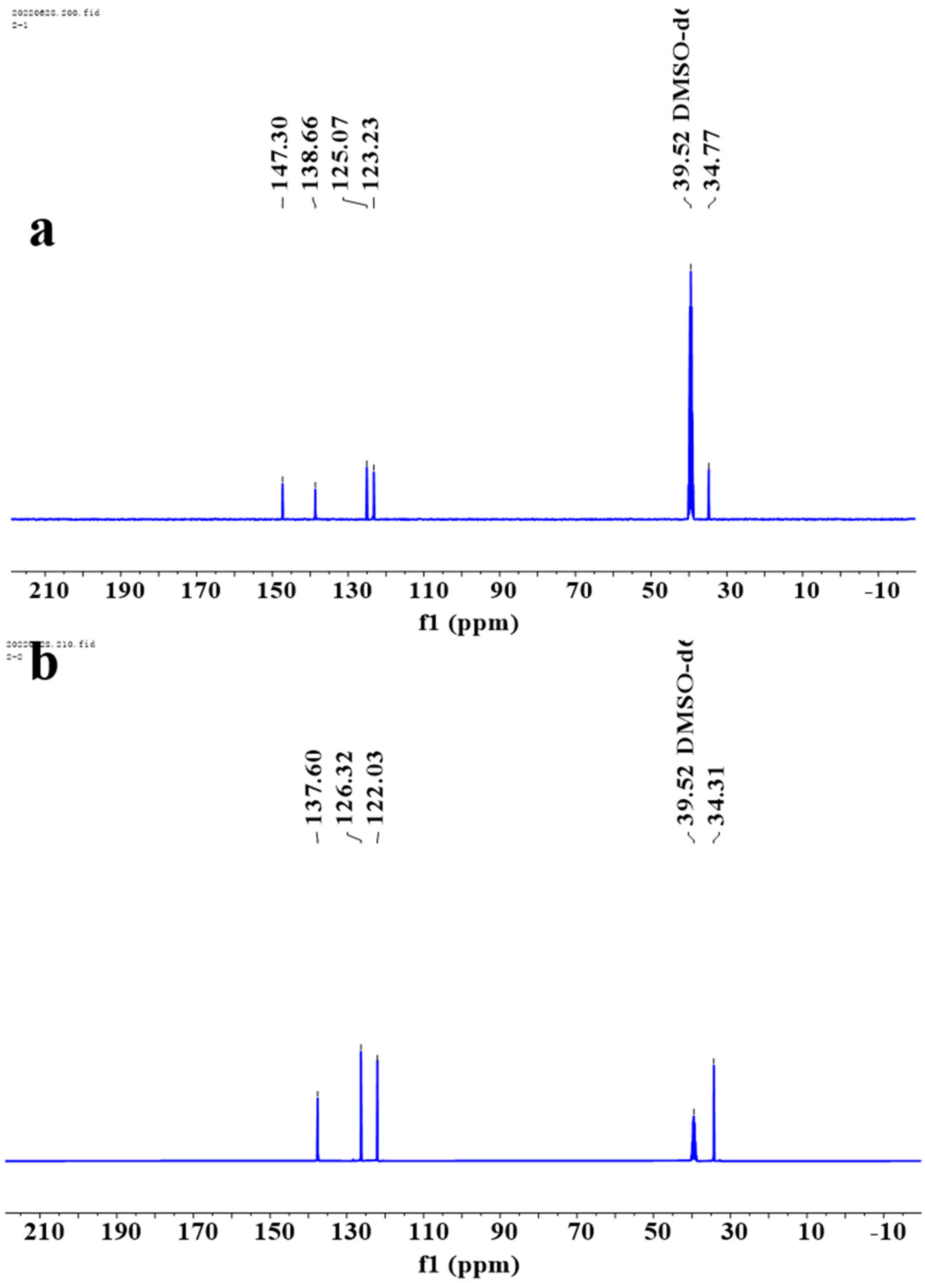

Figure S3. The ^{13}C NMR spectrum of the (1-methylimidazolium)(tetrazol-1-yl)borane (a) and 1-methylimidazole borane (b).

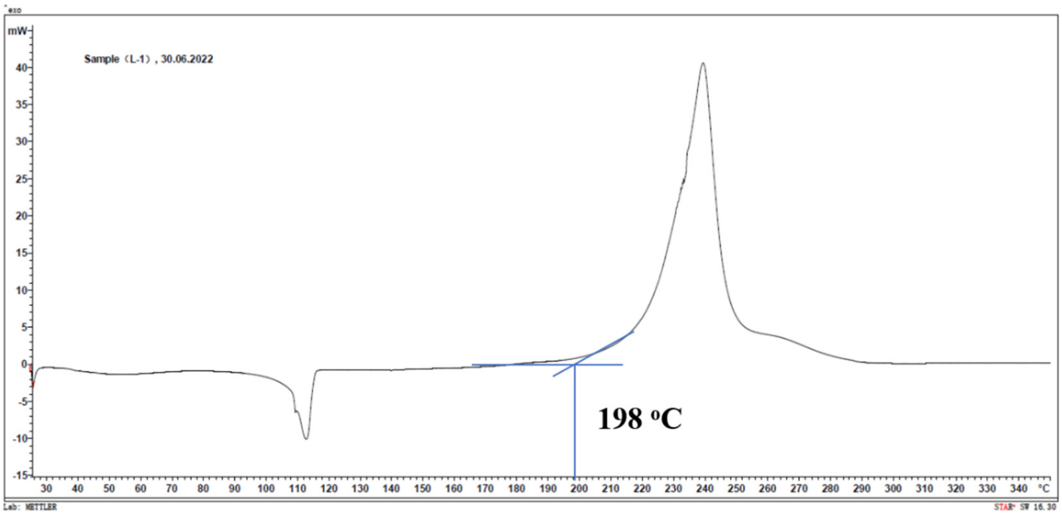

Figure S4. DSC of (1-methylimidazolium)(tetrazol-1-yl)borane.

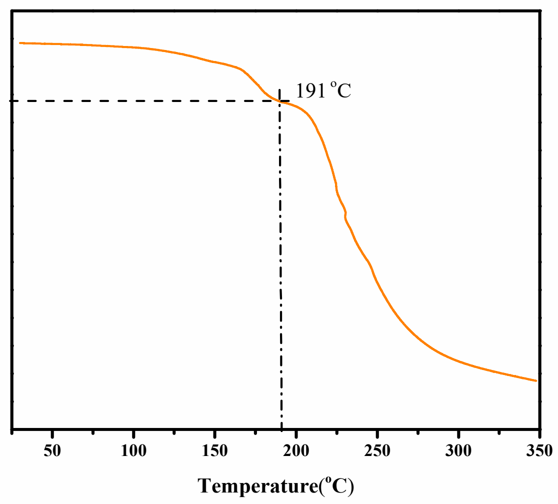

Figure S5. TGA of the (1-methylimidazolium)(tetrazol-1-yl)borane

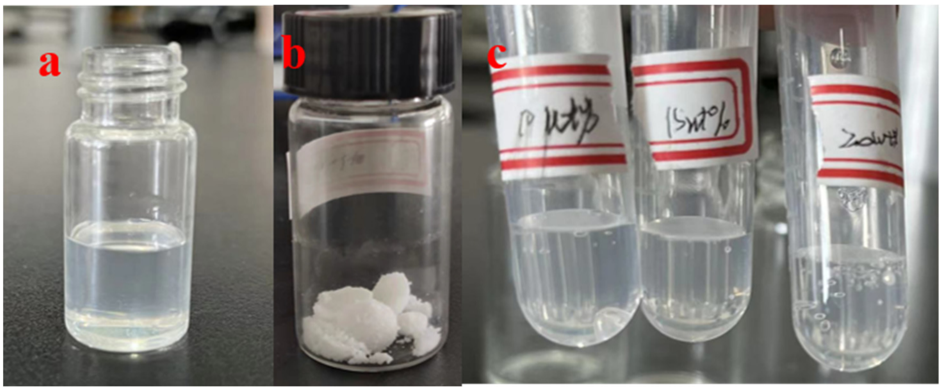

Figure S6. The state of the 1-methylimidazole borane (a), (1-methylimidazolium)(tetrazol-1-yl)borane (b), 1-methylimidazole borane/tetrazole mixtures (10wt%, 15wt%, 20 wt%) (c)

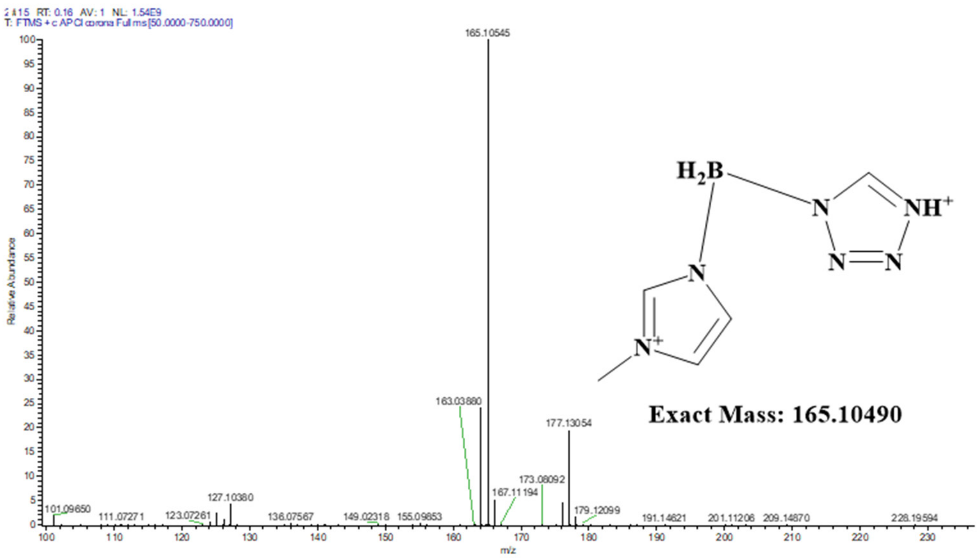

Figure S7. HRMS-APCl spectra of (1-methylimidazolium)(tetrazol-1-yl)borane .

Table S1 Calculated data of gas-phase formation enthalpy based on isodesmic reaction

compounds	E_0 (a.u.)	ZPE (a.u.)	T_C (a.u.)	H_T kJ/mol
NH_3	-56.4154632	0.034373	0.038191	9.62319264
1-methylimidazole	-264.8298954	0.098967	0.105307	15.9798432
Tetrazole	-257.6538579	0.046862	0.051291	11.16320592
(1-methylimidazolium)(tetrazol-1-yl)borane	-547.9234657	0.162053	0.173632	29.18463792
$BH_2NH_2NH_3$	-138.2258063	0.087932	0.094064	15.45558336

Table S2 Gas-phase formation enthalpy data used in the isodesmic reaction

Compounds	ΔH_f(kJ/mol)
1-methylimidazole	127.1[a]
NH_3	-45.9[a]
Tetrazole	321.1[a]
$BH_2NH_2NH_3$	-132.2[b]

[a] from NIST. [b] based on the G2 methods.

Calculation details

Through the Gaussian 09 (Revision D.01) suite of programs, the optimization of structures, frequency analysis, and single energy points were all investigated employing the B3LYP/6-311+G**//MP2/6-311++G**. Based on the isodesmic reactions and G2 methods. However, the result of the ΔH_f was the gas phase enthalpy of formation, while the final products at the room temperature was solid, so the enthalpy of sublimation should be taken into consideration as the equation 1.

$$\Delta H_{sub}=188\ J.mol^{-1}.K^{-1}\times T \quad (1)$$

Based on the results of the ΔH_f, the vacuum specific impulse could be calculated by the NASA-CEA program, where the combustion chamber pressure was 0.95 MPa; area expansion ratio of nozzle was 70; N_2O_4 was chosen as the oxidizer (equivalence ratio =1.0).
